# TEAMs go VR—validating the TEAM in a virtual reality (VR) medical team training

**DOI:** 10.1186/s41077-024-00309-z

**Published:** 2024-09-11

**Authors:** Rafael Wespi, Lukas Schwendimann, Andrea Neher, Tanja Birrenbach, Stefan K. Schauber, Tanja Manser, Thomas C. Sauter, Juliane E. Kämmer

**Affiliations:** 1 Department of Emergency Medicine, Inselspital, Bern University Hospital, University of Bern, Bern, Switzerland; 2 Graduate School for Health Sciences, University of Bern, Bern, Switzerland; 3 Centre for Educational Measurement (CEMO) & Unit for Health Sciences Education, University of Oslo, Oslo, Norway; 4 FHNW School of Applied Psychology, University of Applied Sciences and Arts, Northwestern Switzerland, Olten, Switzerland; 5 Division of Anesthesiology and Intensive Care, Department of Clinical Sciences, Intervention and Technology, Karolinska Institutet, Huddinge, Sweden; 6 Department of Social and Communication Psychology, University of Göttingen, Göttingen, Germany

**Keywords:** Virtual reality, Interprofessional education, Simulation, Nursing student, Medicine student, Medical education, Team training

## Abstract

**Background:**

Inadequate collaboration in healthcare can lead to medical errors, highlighting the importance of interdisciplinary teamwork training. Virtual reality (VR) simulation-based training presents a promising, cost-effective approach. This study evaluates the effectiveness of the Team Emergency Assessment Measure (TEAM) for assessing healthcare student teams in VR environments to improve training methodologies.

**Methods:**

Forty-two medical and nursing students participated in a VR-based neurological emergency scenario as part of an interprofessional team training program. Their performances were assessed using a modified TEAM tool by two trained coders. Reliability, internal consistency, and concurrent validity of the tool were evaluated using intraclass correlation coefficients (ICC) and Cronbach’s alpha.

**Results:**

Rater agreement on TEAM’s leadership, teamwork, and task management domains was high, with ICC values between 0.75 and 0.90. Leadership demonstrated strong internal consistency (Cronbach’s alpha = 0.90), while teamwork and task management showed moderate to acceptable consistency (alpha = 0.78 and 0.72, respectively). Overall, the TEAM tool exhibited high internal consistency (alpha = 0.89) and strong concurrent validity with significant correlations to global performance ratings.

**Conclusion:**

The TEAM tool proved to be a reliable and valid instrument for evaluating team dynamics in VR-based training scenarios. This study highlights VR’s potential in enhancing medical education, especially in remote or distanced learning contexts. It demonstrates a dependable approach for team performance assessment, adding value to VR-based medical training. These findings pave the way for more effective, accessible interdisciplinary team assessments, contributing significantly to the advancement of medical education.

**Supplementary Information:**

The online version contains supplementary material available at 10.1186/s41077-024-00309-z.

## Introduction

Medical errors pose a threat to patient safety and are a serious societal burden [1, 2]. Studies on medical errors have shown that inadequate teamwork is often the cause of mistakes and failures [3]. This is one reason why interprofessional education (IPE) and continuing team training are vital for teaching students and practitioners how to collaborate effectively with other healthcare professions in clinical settings [4–6]. Indeed, an increasing number of studies provide evidence for the positive effects of preparing the healthcare workforce for collaborative practice on health outcomes, attitudes towards IPE [7], and overall patient satisfaction [8, 9]. Thereby, simulation-based training is a well-established method to train teamwork skills [10, 11]. Studies have shown that simulation-based training can improve knowledge, clinical skills, self-efficacy, behaviours, team performance, and clinical practice [12–19]. However, access to simulation-based training and interprofessional education is still limited in most curricula, likely because it is resource-intensive and due to organisational hurdles [20]. Novel, cost-effective alternatives such as virtual reality (VR) simulations could be a way to increase training opportunities [21, 22].

VR, a technology that generates an immersive experience, transporting users to a simulated environment that feels like a different location, by augmenting the primary sensory inputs with machine-generated information [23], is a novel technology and tool for transformative experiences in medical education. VR offers numerous benefits and exerts a positive impact on several aspects of medical education [24, 25]. For example, it enables extensive and iterative training, creating more opportunities for skill development over longer durations [26]. It permits the exploration of complex scenarios with greater frequency, providing learners with invaluable experiences that were previously difficult to replicate. Furthermore, the ability to reset simulations as required generates a robust learning tool, promoting both the correction of errors and the acquisition of skills. Studies indicate that students exposed to VR-based education perform better than peers in traditional settings, demonstrating its effectiveness in enhancing medical learning [27]. VR technology’s enhanced realism and immersion greatly enhance trainees’ confidence and learning experiences [28], leading to improved pass rates and increased student confidence compared to conventional approaches [27]. VR interventions effectively improve self-efficacy, develop skills [29], and acquire knowledge [30, 31]. In addition, users report high acceptability, feasibility, and remarkable emotional impact on the learning experience for VR interventions [28]. Moreover, studies have confirmed the positive impact of VR on attitudes, knowledge, and self-confidence among trainees at different stages [32–34]. However, ensuring fidelity and realism is crucial for obtaining successful outcomes during training, as evidenced by recent research [26, 28]. Additionally, VR technology enables skill training without requiring physical presence, making it particularly advantageous during unprecedented events, such as pandemics [35]. This has the potential to enable practical medical education, which forms the foundation of medical training, even in extraordinary circumstances like a pandemic [36]. Nevertheless, it is also important to note that VR can induce side effects such as cyber sickness [37], and that there is an initial cost [38], which could present a significant barrier to the implementation of VR-based simulation training.

Establishing effective and reliable methods for evaluating team performance is crucial for evaluating the success of a team training, for identifying areas that need improvement and for giving constructive feedback to team members [39, 40]. Yet, at present, no validated tool exists for assessing team performance within VR settings. This deficiency is particularly significant as it hinders the broader adoption of VR for team training and research purposes. Assessing teamwork in VR presents unique challenges. Firstly, it remains uncertain whether the limited expressive capabilities inherent in VR, such as the absence of facial expressions and the constraints on individuals’ body language, allow for effective evaluation of team performance through observational assessment tools. Moreover, the execution of routine procedures and activities in VR may diverge from real-world scenarios, potentially exerting a substantial influence on team dynamics and the means by which they are evaluated.

In principle, various teamwork assessment tools exist [41], such as the Observational Teamwork Assessment for Surgery (OTAS; Undre et al., [42]), the Non-Technical Skills for Surgeons (NOTSS; Jung et al., [43]), the Oxford Non-Technical Skills (NOTECHS; Mishra et al., [44]), the Human Factors Skills for Healthcare Instrument (HuFSHI; [45, 46], and the Team Emergency Assessment Measure (TEAM; [47]. In a recent review [41], in which these tools were compared, the TEAM [47] was highlighted for its uniqueness in analysing the entire interprofessional team as a single unit and for providing a comprehensive solution that allows for effectively capturing the multidimensional aspects of teamwork. In two further reviews, the TEAM was recommended for its reliability and validity [48], and its high methodological quality [49].

The TEAM instrument was conceptualised for resuscitation teams of three or more members [47]. Since its initial publication in 2010, the instrument has been expanded to the assessment of teams in various contexts such as obstetric newborn emergency [50], distributed teams [51], and paediatric emergency [52], as well as in different fields such as pulmonology, neurology, anaesthesiology, surgery, and traumatology [53]. In a recent review, the TEAM’s general validity to assess team performance across hospital clinical teams and in student training was confirmed [54]. Yet, a validation of the TEAM for evaluating team performance in VR-based team training is still pending. To address this gap, we conducted a comprehensive assessment of the reliability and validity of the TEAM instrument within the context of VR team training.

For this purpose, we analysed the TEAM ratings provided by trained observers of an interprofessional team training in VR, featuring pairs of medical and nursing students managing a neurological emergency case [55]. This study not only serves to validate the applicability of the TEAM instrument in VR scenarios but also explores its feasibility in a dyadic setting, particularly in handling an intricate emergency case.

Interprofessional dyads serve as an ideal foundation for training interprofessional teams, representing the smallest unit of such collaboration. These dyads not only enhance learning outcomes in clinical settings compared to uniprofessional groups [56] but also foster a supportive learning atmosphere that aids in achieving clinical objectives [57] and enhance self-confidence of healthcare practitioners [58]. Additionally, it has been demonstrated that dyadic medical training results in lower levels of stress and anxiety among novice participants compared to performing the same training tasks individually, without compromising the quality of performance [59]. Our study thus also provides important insights for trainers and researchers of interprofessional pairs seeking to assess their collaboration.

## Methods

### Study design

This is a prospective validation study with medical and nursing students who completed an emergency medical scenario in VR together as dyadic interprofessional teams. The study took place in May 2023 in the simulation centre of the University Hospital of Bern.

This paper is part of a scientific and educational project, whereby a paper based on data on the acceptance, effectiveness, and feasibility collected during the same interprofessional VR team training will be published elsewhere [56].

### Participants

All sixth year medical students from the University of Bern and third year nursing students from the Bern University of Applied Sciences were eligible to take part in the study. Participation in the study was voluntary and part of an elective course. Inclusion criteria were:

- 18 years of age
- Enrolment as a medical or nursing student, respectively
- Voluntary participation with the signing of the informed consent for the collection and analysis of their personal data in pseudonymised form

Exclusion criteria:

- Epilepsy
- Sensitivity to flashing lights

### Material

#### Demographic survey

Demographic data (age, gender, and study programme) and information on the frequency of taking part in VR simulations and VR games were gathered through an online survey (via www.soscisurvey.de). Respondents used an individually created password so that the gathered information could be combined in pseudonymised form with further data.

#### Scenario and software

The VR scenario employed was developed in-house with the input of emergency medicine professionals and medical education experts. The simulation was a fully immersive supervised VR scenario by SimX Inc. (San Francisco, California, USA). The scenario lasted for about 20 min and displayed a frequent emergency medical issue, namely a patient who was admitted to the emergency department suffering from a severe headache due to an unknown subarachnoid haemorrhage. At a given time, the patient’s condition deteriorated, starting to suffer from an epileptic seizure that required immediate action.

**Fig. 1:**
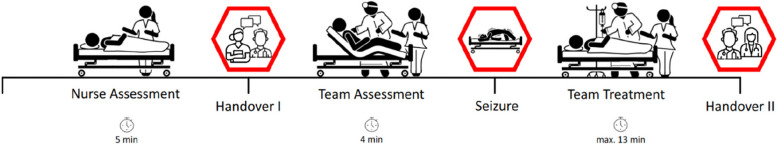
Schematic of the training scenario

In detail, the scenario consisted of three phases (see Fig. 1). During the 5-min “Nurse Assessment”, the student nurse performed an initial triage of the patient in the emergency department. The student nurse was alone in the scenario and took a preliminary medical history and conducted an initial examination (e.g. vital signs). At the end of this phase, the medical student entered the room, and the student nurse conducted a structured handover. During the “Team Assessment”, which lasted for 4 min, the medical student examined the patient jointly with the nursing student. This was followed by a treatment of the patient. The “Team Treatment” commenced after 9 min into the scenario and ended by treatment with benzodiazepine administration or automatically after 7 min. After the correct treatment or elapsed time, the patient was unable to speak for the first 3 min. During this phase, the team could initiate further diagnosis and treatment, followed by either a self-initiated handover to the attending physician or a moderator-triggered handover at the latest after minute 22, which marked the end of the simulation. The simulation was followed by a debriefing.

#### Hardware

To implement a scenario, two Meta Quest 2 VR headsets (Meta Platforms, Inc.; Menlo Park, California, USA) equipped with controllers and noise-cancelling headphones (JBL Tune 760NC, California, USA) were used.

A simulation moderator led through the scenario, using an OMEN gaming laptop from HP (HP Development Company, Bremdalvej 8, 7600 Struer, Denmark). The moderator provided relevant pre-recorded verbal responses and elicited any necessary physiological responses such as lifting an arm from the patient. The moderator had either a background in medicine, nursing, or psychology, and had undergone extensive training in the use of the software.

#### The TEAM 

The TEAM comprises 11 items covering three distinct domains, namely leadership (items 1–2), teamwork (items 3–9), and task management (items 10–11) [47, 54]. Each item is assessed using a five-point Likert scale ranging from 0 (never/hardly ever) to 4 (always/nearly always). Additionally, a global performance assessment (item 12) is included, which captures the evaluator’s overall impression on a scale of 1–10.

Since the TEAM instrument was initially designed for cardiac resuscitation teams consisting of three and more members [47] but the current simulation encompassed pairs and a more complex case with different phases, the standard behavioural markers had to be adapted. This was done by two emergency medicine and simulation experts, a nursing professional, and two psychologists based on two sample videos of good and intermediate team performance during the scenario (Appendix).

#### Procedure

##### Recruitment

**Fig. 2:**
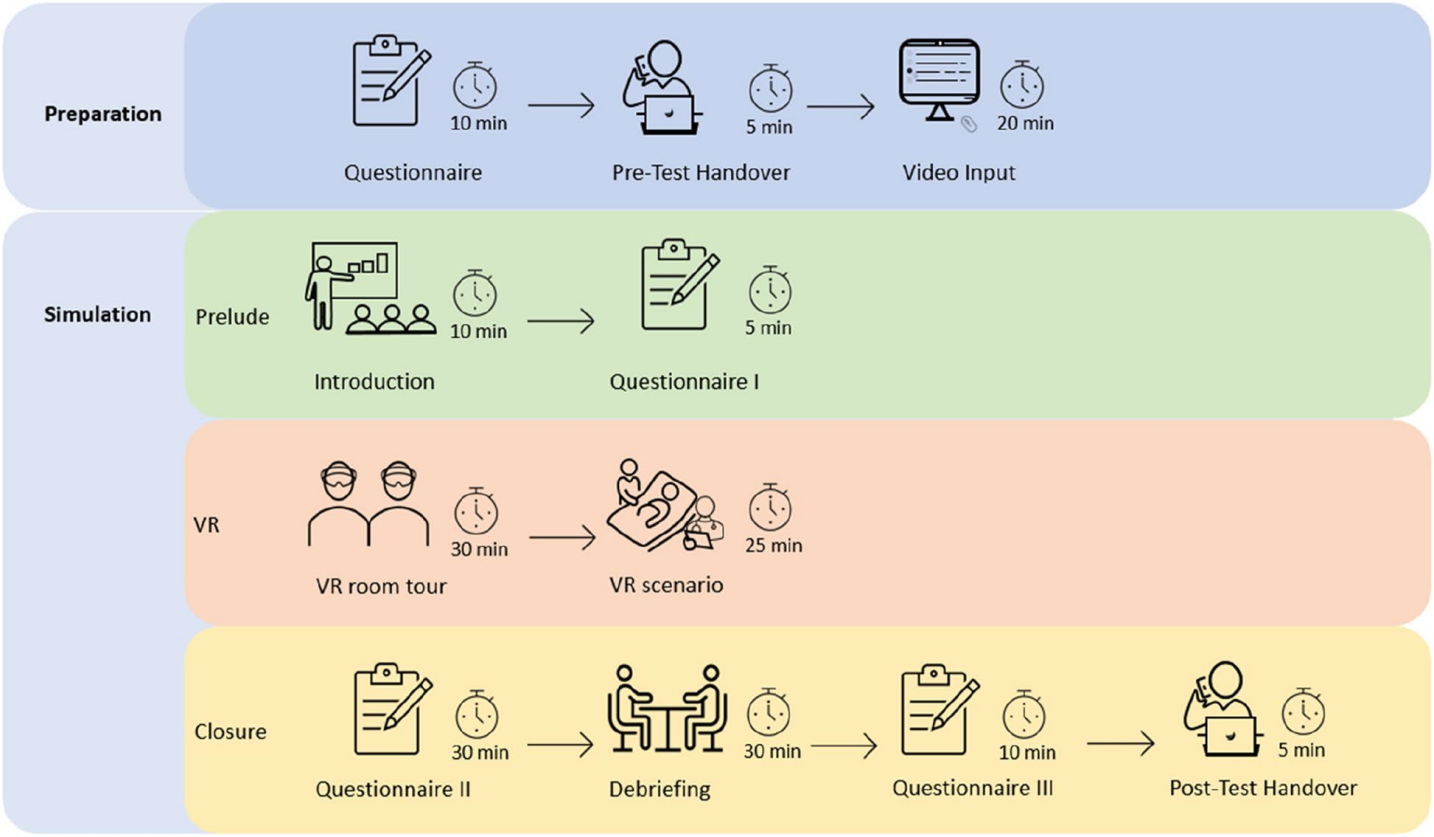
Schematic timetable for the whole time schedule. The training was divided into a preparation and a simulation. The simulation phase was further split into three parts: a prelude, a virtual reality (VR) training, and a closure. The clock adjacent to the corresponding icon represents the duration of each part

All sixth year medical students from the University of Bern and third year nursing students from the Bern University of Applied Sciences were invited via email to take part in an elective university course on emergency medicine (i.e. our study). On a first-come-first-serve basis, they enrolled for the course. They were then assigned to one of three in-person course days. Each day, there were three slots of 3 h with three VR simulations run concurrently. Figure 2 illustrates the procedure from the perspective of a participant, including the time points of different questionnaires that were part of an evaluation study. For more details of the training and evaluation study, please see Neher et al. [55].

##### Preparation

Two weeks prior to the in-person course day, the students were sent a socio-demographic questionnaire, and 1 week prior to the course day, an e-learning video that aimed at refreshing their knowledge on the topics of the simulation (i.e. structured handover, handling a severe headache and epileptic seizures). Additionally, they took part in a pre-test on conducting a handover.

##### Simulation—prelude

The in-person course began with a welcome to all participating students. The simulation team was introduced to the participants, and instructions were provided about the rules and safety precautions for utilising VR. Afterwards, they were randomly paired with a person from the other programme.

##### Simulation—VR

To get acquainted with the VR environment, all pairs received a comprehensive “VR room tour” of the virtual patient’s room. Demonstrations included the use of controllers, the handling of VR objects, and their locations. Ensuring participants’ comfort within the virtual environment was of utmost significance. After answering all questions and a short break, the VR scenario started. Before the student nurse entered the scenario, a briefing on a sheet of paper was presented:

You are a nurse working in a local hospital. You are called to a room in the emergency department to see a new patient. The patient has walked in on his own and has not yet been seen by a physician. Please perform the initial assessment. The physician will soon come to support you. The physician on duty will knock on the door and then support you.

Meanwhile, the medical student was listening to music outside the virtual room, unable to hear or see anything from inside. Following 5 min, the medical student was also given a briefing on a piece of paper:

You are a physician working in a regional hospital. You are called to see a new walk-in patient in the emergency department. The nurse is already there and asks for your assistance. If you hear the knock [of the moderator], you can enter and introduce yourself to the nurse. If you don't get a handover from the nurse, ask for one.

**Fig. 3:**
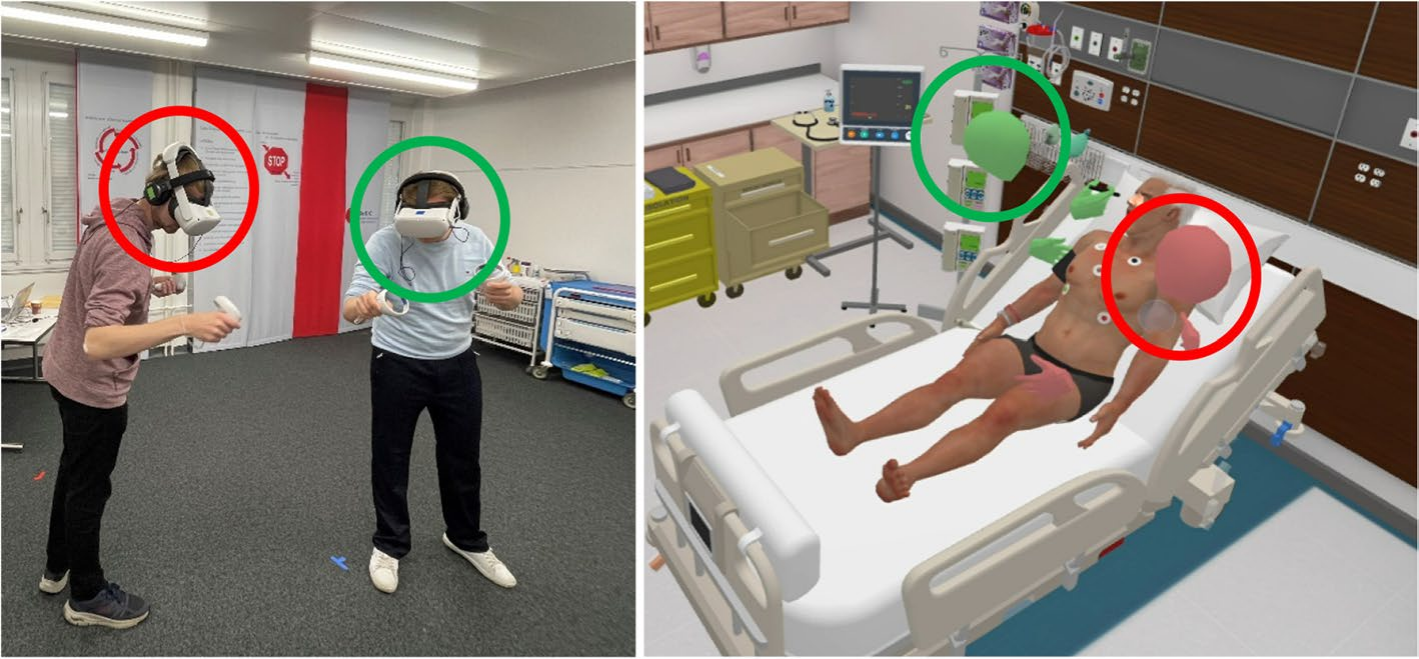
Participants in the VR simulation, **A** in the real training room and **B** in the VR patient room. Note: Red and green circles illustrate the corresponding avatars

The medical student was then directed into the VR simulation under the guidance of a second person, and the rest of the scenario started with a first handover (see Fig. 1), which marks the beginning of the team training that was assessed with the TEAM. The whole simulation was video-recorded in the software from the moderator view. To get an impression of the setup, see Fig. 3.

##### Simulation—closure

At the end of the scenario and after a short break, participants were asked to complete a series of paper questionnaires including various feasibility and usability inventories to evaluate the VR training [56]. This was followed by a debriefing that lasted for about 30 min and focused on the medical treatment as well as interprofessional teamwork.

##### Observers’ rating / data collection

**Fig. 4:**
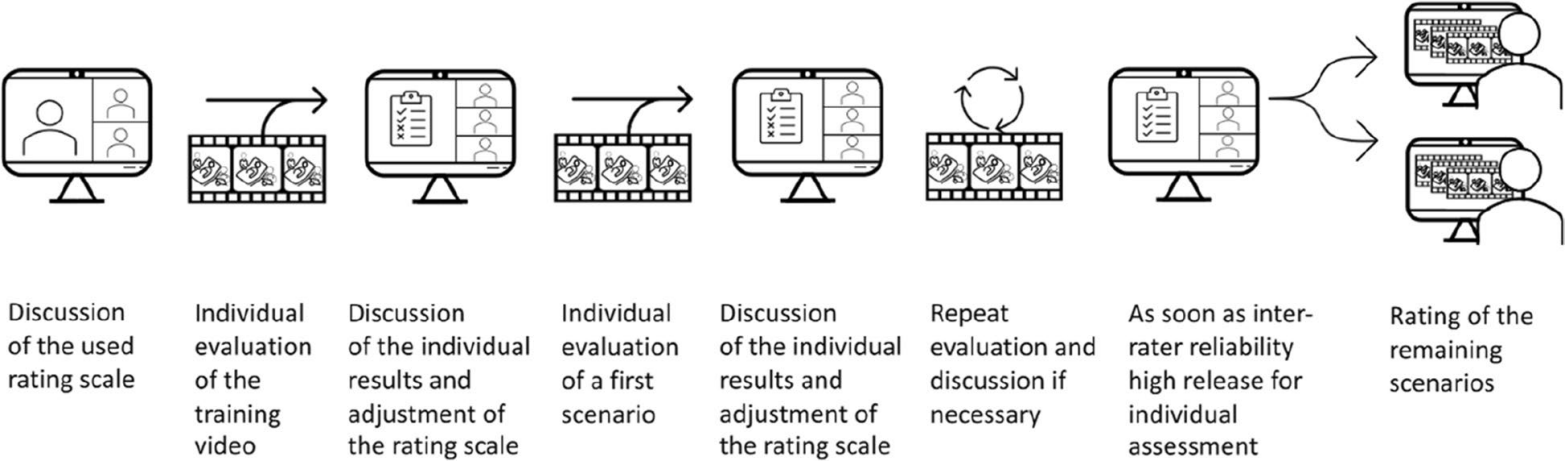
Schematic illustration of the rater training

Two raters out of the study team, one with a background in medicine and nursing (LS) and the other in psychology (RW), were trained by an experienced TEAM user (JEK) in the use of the adapted version of the TEAM instrument. Both raters were actively involved in the planning and implementation of the VR simulation and were therefore familiar with the scenario. Raters were given approximately 15 h of training each, during which they coded one training video and three of the study videos (see Fig. 4). One of the two raters then coded the remaining 17 videos. To estimate the interrater reliability, the other coder independently coded 10 randomly selected videos.

### Data analysis

To compute descriptive statistics and assess the reliability, internal consistency, and concurrent validity, the statistics software “R” (version 4.3.1. R Foundation for Statistical Computing) was used.

For the demographic data, medians, quartiles, and percentages were calculated for the total sample as well as for each study programme subgroup. Mann–Whitney *U* tests were used to calculate whether the two subgroups differed from each other.

Interrater reliability per item was calculated using the *intraclass correlation coefficients (ICC)*, namely the ICC consistency and ICC agreement [60]. ICC consistency reflects the degree to which measurements remain stable across raters, whereas ICC agreement measures the degree of agreement between raters’ ratings. Both ICC consistency and agreement were interpreted using established benchmarks with higher scores indicating a higher level of consistency or agreement between raters [60]: poor (< 0.4), fair (0.4–0.59), good (0.6–0.74), and excellent (≥ 0.75). For further analyses, the ratings of the two observers were averaged if they were not equal.

Internal consistency between all items measuring the same underlying construct (i.e. the TEAM domains leadership, teamwork, and task management) was calculated using *Cronbach’s alpha*, which ranges from 0 to 1. Following established benchmarks [61], we interpreted values as either poor (< 0.6), acceptable (0.6–0.7), good (0.7–0.8), or excellent (≥ 0.8).

*Concurrent validity* evaluates the correlation between two measures or assessments conducted at the same time [62]. Pearson’s correlations were calculated between the global performance score (i.e. item 12) and the three TEAM domains each, with statistical significance established at a *p*-value below 0.05.

#### Ethics

The local ethics committee (Kantonale Ethikkommission Bern) deemed our study to be exempt from full ethical approval, as it is not covered by the Human Research Act (BASEC-Nr: Req-2023–00208). All methods were carried out in accordance with relevant guidelines and regulations.

Written informed consent was obtained (for the training, the video recording, and the data analysis). All data were collected, analysed, and stored in pseudonymised form.

## Results

### Missing data

Initially, 27 time slots were scheduled over a period of 3 days for the VR simulations. Due to non-attendance, one time slot was left vacant, and in five time slots, only one participant appeared so that a member of the study team took the place of the second member during the training. These training sessions were neither recorded nor analysed. In addition, one video was not properly recorded. As a result, a total of 20 teams were analysed.

### Sample

Our sample comprised two groups of participants: nursing students (*N* = 20, 80% females) and medical students (*N* = 20, 50% females), with an average age of 23 and 26 years, respectively (for details, see Table 1). VR experience was reported by 20% of nursing students and 40% of medical students, gaming experience by 50% and 65%, respectively.

**Table 1:** Study population characteristics. *VR* virtual reality, *N* number of participants, *Q1–Q3* quartile 1–quartile 3

	**All participants** *N* = 40	**Nursing students** *N* = 20	**Medicine students** *N* = 20	**Statistics** *p*-value
Age median (Q1–Q3)	25 (23–26)	23 (22–27)	26 (25–28.3)	< 0.001
Female *n* (%)	26 (65)	16 (80)	10 (50)	0.051
VR experience *n* (% yes)	12 (30)	4 (20)	8 (40)	0.251
Gaming experience *n* (% yes)	23 (58)	10 (50)	13 (65)	0.438

### Reliability

The analysis of the interrater reliability using ICC was based on the individual ratings provided by two raters of 10 scenarios. *Agreement* and *consistency* between the ratings were examined for the three TEAM domains.

In terms of *consistency*, the ICC values for the leadership, teamwork, and task management domains were 0.75, 0.90, and 0.77, respectively, indicating an excellent level of consistency. In terms of *agreement*, the ICC values for the leadership, teamwork, and task management domains were 0.76, 0.90, and 0.77, respectively, indicating an excellent level of agreement. Also, the overall rating measured with item 12 shows with a value of 0.91 an excellent level of consistency. For more details, see Table 2.

**Table 2:** Intraclass correlation (ICC) values of each of the 12 items and each of the three TEAM domains rated by two raters. *CI* confidence interval

Items in TEAM	ICC (CI 95%)	ICC (CI 95%)
	Consistency	Agreement
Leadership	0.75 (0.48–0.89)	0.76 (0.49–0.90)
1. The team leader let the team know what was expected of them through direction and command.	0.70 (0.16–0.91)	0.70 (0.19–0.91)
2. The team leader maintained a global perspective.	0.82 (0.43–0.95)	0.84 (0.46–0.96)
Teamwork	0.90 (0.84–0.94)	0.90 (0.84–0.93)
3. The team communicated effectively.	0.84 (0.47–0.96)	0.85 (0.50–0.96)
4. The team worked together to complete the tasks in a timely manner.	0.96 (0.83–0.99)	0.96 (0.84–0.99)
5. The team acted with composure and control.	0.80 (0.38–0.95)	0.80 (0.41–0.95)
6. The team morale was positive.	0.72 (0.21–0.92)	0.74 (0.22–0.93)
7. The team adapted to changing situations.	0.86 (0.55–0.97)	0.85 (0.52–0.96)
8. The team monitored and reassessed the situation.	0.93 (0.74–0.98)	0.93 (0.75–0.98)
9. The team anticipated potential actions.	0.94 (0.77–0.98)	0.94 (0.78–0.98)
Task management	0.77 (0.50–0.90)	0.77 (0.51–0.94)
10. The team prioritised tasks.	0.75 (0.27–0.93)	0.77 (0.30–0.94)
11. The team followed approved standards / guidelines.	0.82 (0.42–0.95)	0.82 (0.45–0.95)
Overall		
12. On a scale of 1–10, give your global rating of the team’s performance.	0.91 (0.70–0.98)	0.91 (0.71–0.98)

### Internal consistency

The internal consistency was calculated using Cronbach’s alpha for the three TEAM domains. Results indicated excellent reliability for the leadership domain (Cronbach's alpha = 0.90) with high correlations among items (*r* = 0.82), good reliability for teamwork (Cronbach's alpha = 0.78) with moderate correlations among items (average inter-item correlation = 0.35), and good reliability for task management (Cronbach’s alpha = 0.72) with moderate correlations among items (average inter-item correlation = 0.62). In addition, we calculated Cronbach’s alpha for the sum of items 1–11, which indicated excellent internal consistency (Cronbach’s alpha = 0.89).

### Concurrent validity

In terms of concurrent validity, Pearson’s correlations between the three TEAM domains and the global performance score (item 12) demonstrated consistently strong associations, with *r* >  = 0.8, highlighting substantial alignment between these domain-specific assessments and the overall team evaluation (*p* < 0.001).

## Discussion

Our study provides evidence for the reliability and validity of the TEAM instrument when assessing the performance of dyadic healthcare student teams engaged in a VR-based team training. Non-technical skills, such as leadership and teamwork skills, play a pivotal role in shaping patient care outcomes and overall teamwork quality [63], and they can be enhanced through appropriate training methods [9]. Amid the ever-evolving landscape of medical education, VR-based training emerges as a transformative tool that has garnered recognition from numerous researchers and experts in the field [64]. It offers distinct advantages, such as location independence, cost-effectiveness, and broader accessibility, effectively surmounting the limitations associated with traditional physical simulation training [21]. Through our study, we offer valuable insights to benefit trainers, researchers, and developers of VR-based training, expanding the evidence supporting the applicability of the TEAM for this innovative approach.

Our assessment of interrater reliability for the TEAM instrument demonstrated excellent consistency and agreement across all three domains when evaluated by two proficient raters based on video recordings of the VR simulations. Notably, these results were achieved despite the inherent limitations of VR settings for observational assessment, which include reduced (or in our case: no) capacity to capture facial expressions, gestures, and gaze direction. Nevertheless, it is worth considering that these limitations may have a more pronounced impact on interrater reliability when dealing with larger team sizes as opposed to our dyadic setting.

In our examination of the internal consistency and concurrent validity of the TEAM instrument, we observed strong evidence for good to excellent consistency and substantial congruence between the domain-specific assessments and the overall team evaluation. This replicates previous research conducted in real-life training scenarios (e.g. Freytag et al., [53]) and various contexts (e.g. Morian et al., [51]). Importantly, our adaptation of the behavioural anchors for the TEAM items to suit our specific intricate emergency scenario and a dyadic team setting did not compromise the robustness of these findings. This emphasises the flexibility and adaptability of the TEAM instrument to various training environments.

Our study’s validation of the TEAM instrument in a virtual reality (VR) scenario addresses a need for healthcare education in the near future, in which the ability to accurately assess and improve teamwork skills is paramount. Having a valid and reliable measurement tool like the TEAM instrument ensures that evaluations are robust and meaningful. This, in turn, enables educators to tailor training programmes effectively, maximising the benefits for every learner. Non-technical skills are often overlooked but have a profound impact on patient care quality. Measuring team performance is also an essential step in research for understanding the complex factors that contribute to successful teamwork dynamics [65]. The use of precise evaluation tools, like the validated TEAM instrument, is necessary to identify areas for improvement and guide targeted training. This ensures that healthcare professionals are well-equipped to navigate the complexities of their roles, ultimately benefiting patient safety and overall healthcare effectiveness.

### Limitations

Our study comes with the limitation that it is a single-centre study with a moderately sized sample. While our findings and sample sizes align with those of comparable studies [47, 66], it needs to be acknowledged that the study involved 20 teams within one specific scenario, and our results stem from assessments conducted by two raters, although more would be desirable [60]. Whether our results can be extrapolated to other VR scenarios and larger teams, where the limitations of VR observations might be more pronounced, remains a topic for future research. Moreover, the limited addressal of validity’s complexity and insufficient use of broad validation frameworks in health professions education might affect the broader applicability of our study’s findings [67, 68].

### Conclusions and future directions

VR-based training presents substantial advantages for medical education, particularly in the context of team training. We have demonstrated that the TEAM instrument is well-suited for reliably evaluating team performance within an interprofessional VR-based team training scenario. Nonetheless, it is important to recognise that observational tools like the TEAM have their own limitations (such as capturing only observable behaviour but no inner states). Yet, they can be complemented by objective measures such as electrocardiogram (ECG), electrodermal activity (EDA), and eye-tracking, which may allow providing a more comprehensive assessment of team performance. While this data-driven approach is not without its limitations [64], it offers the potential for objective insights into team dynamics [69–71]. Investigating effective ways to integrate these diverse approaches should be a focus of future research.

## Supplementary Information

Additional file 1: Appendix.

## Data Availability

Requests to access the datasets should be directed to RW, rafael.wespi@extern.insel.ch.

## Abbreviations

ECG: Electrocardiogram
EDA: Electrodermal activity
ICC: Intraclass correlation coefficient
TEAM: Team Emergency Assessment Measure
VR: Virtual reality
