# Supplementary material for: TEAMs go VR—validating the TEAM in a virtual reality (VR) medical team training
Source: Adv Simul (Lond). 2024 Sep 11;9:38. doi: 10.1186/s41077-024-00309-z (PMC11389291; doi:10.1186/s41077-024-00309-z)
Supplement: Supplementary file 1 — Additional file 1: Appendix. [file 41077_2024_309_MOESM1_ESM.docx]

| **Leadership: It is assumed that the leader is either designated, has emerged, or is the most senior – if no leader emerges allocate a “0” to Questions 1 & 2.** | |
| --- | --- |
| 1. **Team leader let the team know what was expected of them through direction and command** | |
| **0= Never/Hardly Ever** | **4= Always/Nearly Always** |
| - Not identifiable - Does not delegate roles or distribute workload - Does not articulate or share team goals clearly - Does not explicitly articulate or establish priorities | - Clearly identifiable as the Team Leader - Clearly distributes workload - Clearly articulates or shares team goals - Explicitly articulates or establishes priorities |
| 1. **The team leader maintained a global perspective**   *Prompts: Monitoring clinical procedures and the environment? Appropriate delegation?* | |
| Team Leader:   - Does not share thought process - Does not summarize or recap events for the team | Team Leader:   - Shares thought process; makes thinking transparent (i.e., speak out loud) - Summarizes and recaps events for the team - Collect missing information |

| **Team Work: Ratings should include the team as a whole (i.e., the leader and the team as a collective (to a greater or lesser extent)).** | |
| --- | --- |
| 1. **The team communicated effectively**   *Prompts: Verbal, non-verbal and written forms of communication?* | |
| **0= Never/Hardly Ever** | **4= Always/Nearly Always** |
| - Orders are complex and difficult to understand (e.g., several orders are given at once, non-standardized format, information missing) - Communication is not purposeful (i.e., much extraneous talking, communication does not contribute to achieving team goals or priorities) - No observable closed loop communication - Confusion is not clarified (confusion due to technical problems are not counted) | - Orders are complete and given in a standardized or clear format - Communication is clear, concise, easily understood, and purposeful - Consistent use of closed loop communication (i.e., person receiving message confirms that it has been received and gives appropriate read-back) - Any confusion is clarified (confusion due to technical problems are not counted) |
| 1. **The team worked together to complete tasks in a timely manner** | |
| - Team appears inefficient and unorganized - No evidence of coordination of activity - Both, leader and team member, doing same task, leaving other tasks unattended - Task saturation goes unnoticed by team members; team members do not offer support or help | - Team appears efficient and organized (i.e., a “well-oiled machine”) - Evidence of individual coordinating activity (e.g., distributing and delegating tasks) - Work is distributed amongst the team - Team members around the bed are engaged in purposeful activity (i.e., team members who are not needed are directed away from the bed) - When task saturation of a team member is noted, other team members offers support or help |
| 1. **The team acted with composure and control**   *Prompts: Applicable emotions? Conflict management issues? Psychological safety?* | |
| - Atmosphere appears chaotic - Team members’ voices are raised; yelling, swearing - If conflict arises, there is long discussion rather than respectful and efficient resolution | - Atmosphere appears organized, calm, controlled and professional - Team members exhibit calm tones of voices - Alternating points of view are expressed and resolved respectfully and efficiently |
| 1. **Team morale was positive**   *Prompts: Appropriate support, spirit, optimism, determination?* | |
| - Team members do not assist one another with tasks - Team members exhibit harsh and abrupt tone of voice - Team members criticize each other | - Team members assist one another with tasks - Team members exhibit collaborative, team-oriented behavior - Team members express their viewpoints in a respectful way |
| 1. **The team adapted to changing situations**   *Prompts: Adaptation within the roles of their profession?Situation changes: Patient deterioration? Team changes?* | |
| - Team members are not made aware of changing situation (i.e., when situation changes, no one reports the change to the other or it is noticed significantly late) - Team members fixate on prior tasks and don’t adapt to changing situation; new tasks remain unassigned - In response to changing situations, team does not adapt or change focus to accommodate | - When situation changes, team member(s) report to the other in a timely fashion (i.e., change in patient hemodynamic stability, change of team roles, change of plan of care) - Team roles change dynamically or may be reassigned within their profession because of change in team composition or to meet shifting priorities (e.g., O_2_ sats falling, significant rhythm change on monitor, drop in blood pressure, change in mental status)   In response to changing situations, team adapts and changes focus to accommodate |
| 1. **The team monitored and reassessed the situation** | |
| - No verbalized reassessment of patient or situation - Vital signs are not called out - Team members do not exhibit situational awareness; do not verbalize changes to the team   No evidence of shared mental model (i.e., no team members share thinking regarding the patient status) | - Team members continually reassess patient and trends or changes are verbalized (i.e., improvement or deterioration of patient status) - Vital signs are called out - Team members exhibit situational awareness; verbalize changes to the team   Evidence of shared mental model (i.e., team members share thinking regarding the patient status) |
| 1. **The team anticipated potential actions**   *Prompts: preparation of defibrillator, drugs, airway equipment?* | |
| - Team is playing “catch up” (i.e., significant delay in critical intervention because team was not prepared - e.g. airway equipment, meds)   No team member verbalizes concern for potential changes in situation | - As clinical situation allows, meds, airway equipment are anticipated and planned for use (e.g., potential for respiratory failure exists, team prepares airway equipment and draws up meds)   Team member verbalizes concern for potential changes in situation |

| **Task Management** | |
| --- | --- |
| 1. **The team prioritised tasks** | |
| **0= Never/Hardly Ever** | **4= Always/Nearly Always** |
| - Team is doing unnecessary activities (i.e., wrong medication) - Priorities are not clear - Critical tasks remain undone or not completed (e.g., O_2_ sats are falling and team member(s) remain focused on putting in a Foley catheter) | - Team is engaged in necessary activities - Priorities are clear and team is focused on tasks to achieve those goals - Prioritized activity to care for the patient to achieve value-added outcomes |
| 1. **The team followed approved standards/guidelines**   *Prompts: Some deviation may be appropriate?* | |
| - Significant deviation from critical elements and guidelines - Many items need improvement | - Adherence to appropriate standards and guidelines (i.e., ISBAR, pain assessment, ABCDE) - Few items need improvement |

| **Overall** | | |
| --- | --- | --- |
| 1. **On a scale of 1-10 give your global rating of the team’s performance** | | |
| **1-3** | **4-7** | **8-10** |
| - Team is dangerous - Team exhibits activities that result in significant safety concerns | - Team appears competent overall - Some areas of improvement needed - Team gets the job done | - Excellent, a model of good teamwork - Not many areas of improvement |
